# Cell proliferation and carcinogenesis: an approach to screening for potential human carcinogens

**DOI:** 10.3389/fonc.2024.1394584

**Published:** 2024-05-28

**Authors:** Samuel M. Cohen

**Affiliations:** Havlik-Wall Professor of Oncology, Department of Pathology, Microbiology, and Immunology and the Buffett Cancer Center, University of Nebraska Medical Center, Omaha, NE, United States

**Keywords:** two-year bioassay, mutagenesis, immunosuppression, estrogenic activity, cytotoxicity, regenerative proliferation, cell proliferation

## Abstract

Cancer arises from multiple genetic errors occurring in a single stem cell (clonality). Every time DNA replicates, mistakes occur. Thus, agents can increase the risk of cancer either by directly damaging DNA (DNA-reactive carcinogens) or increasing the number of DNA replications (increased cell proliferation). Increased cell proliferation can be achieved either by direct mitogenesis or cytotoxicity with regenerative proliferation. Human carcinogens have a mode of action of DNA reactivity, immunomodulation (mostly immunosuppression), increased estrogenic activity (mitogenesis), or cytotoxicity and regeneration. By focusing on screening for these four effects utilizing *in silico*, *in vitro*, and short-term *in vivo* assays, a biologically based screening for human chemical carcinogens can be accomplished with greater predictivity than the traditional 2-year bioassay with considerably less cost, less time, and the use of fewer animals.

## Introduction

Means to discover chemicals that can increase the risk of cancer in humans has been a goal of science for over a century. Approaches have involved the development of epidemiology studies of various populations, beginning originally with studies of various occupational settings but more recently utilizing investigations of broad populations. The second means of screening for carcinogens is to evaluate the chemicals in animal models, most notably the long-term rodent bioassays in rats and mice, which have evolved since the 1960s. Considerable concern has arisen over the years about the performance of such studies so that efforts are now underway to develop alternative tests to screen for chemical carcinogenicity. Furthermore, efforts are being made to reduce the number of animals utilized in toxicologic evaluations.

Concerns regarding the long-term rodent bioassays have extensively been described in the literature and include the high cost, the long time to perform such assays, the use of large numbers of animals, and the interpretation of the assays, but most notably there is increasing realization that the results of the long-term bioassay in rodents are frequently not predictive of effects in humans (1–5). Any time an experiment is performed in animal models, two basic assumptions are made, namely: (1) what happens in the animal model will also happen in humans (species extrapolation) and (2) the response observed at the doses used in the animal model will be relevant to the exposure levels in humans (dose extrapolation). For some chemicals, these assumptions may be reasonable; however, for many chemicals, one or both of these assumptions are incorrect.

Lack of relevance of the animal findings to humans includes such examples as α_2_u-globulin as related to kidney tumors in male rats (6), PPARα activator-related liver tumors in rats and mice (7, 8), urinary bladder tumors secondary to the administration of high doses of various sodium salts (saccharin, ascorbate, and bicarbonate) inducing urinary bladder tumors in rats (6), and statins producing liver tumors in rats and mice (9–11). Numerous examples have likewise been identified of tumors being produced in animal models at a high dose that are not relevant to human exposures at lower levels, with a prototypic example being chloroform-induced liver and kidney toxicity and tumors (12). Humans exposed to high doses of chloroform also produce liver and kidney toxicity, but environmental exposures in the drinking water are at concentrations several orders of magnitude less and do not produce cytotoxicity. They are therefore not considered relevant to human cancer risk at human exposure levels. The presence of thresholds have been clearly demonstrated for non-genotoxic carcinogens, and there is increasing evidence that even for genotoxic carcinogens there is a threshold (13–17). Exposure to levels below these thresholds would not increase cancer risk.

There have been many attempts to identify alternative tests for screening for carcinogenicity for chemicals, beginning with the development of the Ames genotoxicity assay utilizing *Salmonella* bacteria (18, 19). Although numerous other examples have been developed for specific types of tumors, there remains no clear approach utilizing alternative methods to evaluate carcinogenicity that is accepted both scientifically and in regulatory settings.

Nevertheless, considerable effort is being made to develop alternative approaches. This includes the approach being taken by the pharmaceutical industry as illustrated in the new ICH (20) guidelines that have been developed to provide guidance for the kinds of data that can be used to provide a weight of evidence evaluation that would preclude the necessity of performing long-term bioassays. Waiver programs have likewise been developed in various agencies, including the US Environmental Protection Agency (21) and the European Chemical Agency (ECHA), attempting to reduce the reliance on animal testing for carcinogenicity. The focus of these new approaches is entirely based on the mode of action considerations.

The present approach to carcinogenicity screening utilizing animal models is to perform the long-term assay, identify any tumors that are increased in incidence in rats and/or mice, and then evaluate whether the mode of action and/or the dose is relevant to human exposures. A framework for the evaluation of mode of action and human relevance of toxic endpoints has been developed by the International Programme on Chemical Safety (IPCS) and by the US EPA and Health Canada (22–26). This has been incorporated into regulatory guidelines and has evolved to the development of adverse outcome pathways (AOP) (27, 28). The newer alternative approaches start with the idea of evaluating various modes of action in short-term *in vivo* and *in vitro* assays, with long-term animal testing not being required. Various approaches have been described based on the mode of action (4, 5), and this paper describes an elaboration of that approach.

## Basic principles of carcinogenesis

Utilizing a mode of action approach, we first have to develop a basic understanding of carcinogenesis. Research over the past century has clearly demonstrated that cancer arises due to multiple genetic errors occurring in the stem cell population of a given tissue (4, 5, 29, 30). More than one error is required, although how many errors are actually required for individual tumors is generally not definitively known at this time. All of the errors in the DNA must accumulate in a single cell since cancer is considered a clonal disease. Furthermore, carcinogenesis is considered a probabilistic (stochastic) process, that is, it is not whether exposure to chemical X actually leads to tumor Y, but rather whether the level of exposure to X has a certain probability of leading to the development of tumor Y. In cancer, the mistakes have to occur in the genes that are critical to the development of a given cancer and have to be in a portion of that gene that is relevant to its function or control of its expression. Lastly, it is well known that every time DNA replicates those mistakes occur, albeit uncommonly; nevertheless, mistakes occur every time DNA replicates.

Based on these considerations, a chemical can increase the risk of cancer in one of two basic ways, namely: (1) the chemical can damage DNA directly so that more mistakes are made every time DNA replicates or (2) the agent can increase the number of DNA replications, providing more opportunities for critical mistakes to occur in the critical genes, leading to the development of cancer (29–31). These effects on toxicity or cell proliferation can be direct or indirect, frequently require metabolic activation of a chemical, and can be secondary to the activation of other systems such as immunomodulation, leading to the increased expression of various viruses which are known to be oncogenic (32, 33).

Numerous metabolic activation processes have been identified for DNA-reactive carcinogens, which have been well delineated in the literature. It is this process that is best screened for by the Ames Assay and other mutagenesis assays as well as computerized Structure Activity Relationships (SAR) (34).

Numerous processes have likewise been identified by which increased DNA replication can be produced (35, 36). It is important to recognize that this is the number of DNA replications and not necessarily the rate, although they frequently go together. However, there has been a misunderstanding in the literature that increased cell proliferation is recognized only by an increase in rate, usually determined by labeling indices such as bromodeoxyuridine (BrdU) or Ki-67 immunohistochemical methods. This issue is particularly notable in the gastrointestinal tract where the stem cell population is already proliferating at a high rate, and increased proliferation is generally not reflected by an increase in rate but rather an accumulation of the appropriate stem cell population (such as the crypts of the intestine) (37).

Increased cell proliferation can be caused by either an increase in cell births or decrease in cell deaths, which leads to an accumulation of more cells (31, 38, 39). Increased cell births can be produced either by direct mitogenesis (directly inducing cells to replicate) or, more commonly, by cytotoxicity (cell death) with consequent regenerative proliferation. Decreased cell deaths can be produced either by increasing apoptosis in certain tissues or decreasing cell differentiation, which is a cell death process. More than one of these processes may be present for a given chemical. DNA-reactive carcinogens if administered at high enough doses will also produce cytotoxicity with regenerative proliferation, leading to a synergistic effect. This has been illustrated with the carcinogen 2-acetylaminofluorene (2-AAF) for the development of liver and urinary bladder tumors in mice in the so-called megamouse (ED_01_) study performed at the National Center for Toxicological Research in the 1970s and modeled by Ellwein and Cohen in the 1990s showing the interaction between DNA damage and increased cell proliferation (40).

## Modes of action of human carcinogens

Although numerous chemicals and other agents have been identified as causing cancer in humans, fundamentally they all act by one of four basic modes of action, namely: (1) DNA-reactive mutagenesis, (2) immunosuppression, (3) increased estrogenic activity, and (4) cytotoxicity and consequent regenerative increased cell proliferation. Multiple examples of these have been identified in the human population.

A variety of classes of mutagenic carcinogens have been identified in the past century, beginning with polycyclic aromatic hydrocarbons (PAH) and subsequently including such agents as aromatic amines, N-nitrosamines, aflatoxins, phosphoramide mustards, aristolochic acids, and other agents. Such chemicals are usually positive in the Ames Assay, particularly if an appropriate metabolic activating system is utilized (18, 19). It is important to note that just because a chemical produces a DNA adduct does not mean that it necessarily will be mutagenic and therefore not necessarily carcinogenic (13, 14). The adduct has to be at a site that is involved in base pairing or can be shown to produce apurinic or apyrimidinic sites. These chemicals are also usually positive in the long-term rodent bioassay.

Immunosuppression or, more accurately, immunomodulation is also well known to be the basis for human carcinogenesis (32, 33). Immunosuppression can be produced in humans either by an inherited disorder, secondary to treatment with pharmaceuticals used for transplantation or for the treatment of various diseases such as autoimmune disorders or various cancers, or by AIDS. Regardless of the cause of the immunosuppression, there is an increased risk for the development of cancer. However, it is not an increased risk of all cancers; rather, it is an increased risk mostly of tumors related to the activation of various oncogenic viruses such as Epstein–Barr virus (EBV), human papilloma virus (HPV), or Kaposi’s sarcoma virus (KSV, also known has human herpes virus 8, HHV8) and possibly others. Also increased are certain other tumors such as melanoma, which has a high mutation rate and presumably generates neoantigens. Kidney transplant patients also develop an increased risk of kidney cancers due to their chronic kidney disease since kidneys usually are retained in the patient in whom the transplant is placed. Chronic kidney disease is a well-known cause of increased risk of renal tubular tumors (41). A major difficulty with the long-term rodent bioassay is the fact that many immunosuppressive agents are actually negative in that assay whereas they are well known to cause cancer in humans (42). Thus, the animal bioassay produces false negative results, the worst outcome for any screening assay. False positives can be dealt with, but false negatives are a serious issue.

Increased estrogenic activity is also the cause of certain tumors in humans, most notably breast cancer and endometrial cancer but also including uncommon liver hepatocellular tumors and possibly tumors at other sites (43). This is generally reflected as not just an increase in estrogen but also an imbalance between estrogen and progesterone. Estrogen-related carcinogenesis is related to the mitogenic effect of the interaction of estrogen and estrogen-like chemicals with estrogen receptors, but there is evidence that for at least the liver and possibly the breast, those DNA adducts that form from estrogen metabolites might also play a role (44). Estrogen and estrogenic chemicals also produce tumors in animal models, but dose is a critical consideration.

Lastly, there is the mode of action of cytotoxicity and consequent regeneration. This is a common mode of action in animal models for non-genotoxic chemicals, such as chloroform as mentioned above (12), but has been clearly associated with chemicals in humans (4). Arsenic is one example (45). This mode of action clearly shows a threshold as part of the dose response, and frequently the dose is quite high in animals compared to exposures in humans, with the example being chloroform again.

## Screening for human carcinogens

Since human carcinogens act by one or more of these four modes of action, screening for human carcinogens can be based on screening for these four modes of action, much of which can be accomplished with assays that are already available, many of which are *in vitro* but not all (4, 5).

Screening for mutagenesis is the most developed of the four modes of action. This involves essentially performance of the Ames Assay with appropriate metabolic activating systems (18, 19). Chemicals involving indirect genotoxicity, such as chromosomal aberrations and micronucleus formation, are not strictly appropriate for this determination. To begin with, these latter assays rely on cytotoxicity for the results, are indirect, and involve thresholds (46–49). Furthermore, it is not clear that these assays are strictly predictive of carcinogenicity rather than indirect genotoxicity being related to cytotoxicity. Computational models for mutagenicity are well developed and can be utilized in conjunction with the Ames Assay (34). A problem with the chromosomal aberration and micronucleus *in vitro* assays is their propensity to produce false positives. There are numerous examples of false positives in the *in vitro* assay when they are evaluated in various *in vivo* assays (46). *In vivo* evaluation of direct mutagenesis detected in the Ames Assay can also be performed, such as the MutaMouse or Big Blue. In reality, a chemical being developed for commercial use that gives a positive result in an Ames Assay will generally not be developed for commercial use unless there is a strong benefit, such as use in cancer chemotherapy.

Assays for immunotoxicity have also been well developed. It should be realized that the use of mouse models, in particular, are not very useful in predicting human immunomodulation effects (50), and as noted above, screening for immunosuppression in animal models is not particularly useful in screening for their carcinogenic activity (42). This is partly due to the fact that mice have a high background of oncogenic retroviruses incorporated in their genome so that production of lymphohematopoietic neoplasms in mice are not particularly relevant to humans (51, 52). The production of the so-called splenic mononuclear cell leukemia (MCL) in rats is likewise primarily found in the F344 rat strain, and it does not appear to be relevant to human cancer risk (53).

*In vivo* evaluation of immune effects can be performed in rodents, but their relevance to humans is questionable (50). Nevertheless, evaluation in short-term assays for an effect on lymphoid tissues can be performed, including evaluation of hematologic parameters and examination of lymphoid tissues such as thymus, lymph nodes, spleen, mucosa-associated lymphoid tissue (MALT), and bone marrow, which can be performed in animals. However, more useful is a direct evaluation in primary human lymphoid cells which are readily obtainable from human donor samples. These can be utilized for the evaluation of the numerous complex responses of the immune system, including the multiple cell types (B cells, T cells, NK cells, etc.) and for the evaluation of various cytokines. Such an evaluation is already available, albeit requiring considerable resources. Nevertheless, it avoids utilization of animals and is evaluating the response in human cells, not rodent cells.

The difficulty with evaluating effects *in vitro*, whether immune or other effects, involves numerous variables, most of which have not been adequately addressed. To begin with, metabolic activating systems are necessary or the chemical and its potential metabolites have to be evaluated. There are also issues as to whether to utilize immortalized cells or primary cells. Immortalized cells pose considerable difficulty for interpretation since they are already abnormal with numerous genetic abnormalities, commonly including karyotypic abnormalities (54). Utilizing primary cells is preferred, but that raises the question as to which primary cells to use and how many different donors are to be evaluated. Considerations include the age of donors, sex, race, and possibly other variables. How many different primary cell donors need to be evaluated to adequately screen for the human population? This is a significant issue not only for the evaluation of the immune system but for any other parameter in screening. Considerable effort should be made in addressing this issue.

Evaluation of estrogenic activity is already being performed (55) utilizing a variety of *in vitro* systems, including binding and activation of estrogen receptors as well as responses to estrogen activation. If necessary, *in vivo* systems are also available, such as the uterotrophic assay (56). A major consideration in evaluating estrogenic activity is dose.

In the pharmaceutical industry, it has become apparent that there are certain classes of drugs which are known to increase cancer risk because of the receptor target (57). Generally, these involve direct mitogenic effects or cytotoxicity and regenerative effects and are related to the molecular biologic response of the target. Screening for off-site target interactions can be performed by utilizing a variety of *in vitro* receptor-mediated assays. These are generally performed at a dose up to a maximum of 10 μm. This raises the entire issue of what doses are to be used and what limits for any *in vitro* assay. In general, a maximum of 10× or 100× the human exposure level (blood, urine, or other appropriate fluid) should be set. This becomes particularly important when evaluating cytotoxicity since all chemicals can be cytotoxic if a large-enough concentration is utilized in the *in vitro* assays. Certain ranges need to be established as to what is relevant for human exposures. A result in an assay at a high concentration that is positive needs to be put into perspective regarding the human exposure, taking into account thresholds.

The mode of action involving cytotoxicity and regenerative proliferation poses a major challenge for utilizing new methodologies (35, 36). At the present time, this requires *in vivo* screening in animal models, not just in rodents since relevance needs to be evaluated in other larger animal species such as dogs, non-human primates, or mini pigs. Evaluation *in vivo* needs to be made for evidence of increased cell proliferation as well as increased toxicity. This includes evaluation of various markers of cytotoxicity such as hematologic parameters, liver enzymes and kidney markers in blood, and evaluation of various tissues for toxicity and/or increased cell proliferation. Proliferation can be indicated by the presence of hyperplasia in various tissues, but not all. Most notably, some evaluation of DNA replication (labeling index) needs to be made since that is a more sensitive marker for increased proliferation than histopathology is (58, 59). However, not all rodent tissues need to be examined for both cytotoxicity and increased proliferation to screen for human carcinogens. This is because a number of tissues in rodents are not indicative of cancer in humans (**Table 1**) (2, 60). These include tissues that are present in rodents but not present in humans (forestomach, Harderian gland, and Zymbal’s gland) or tumors that occur in animals that do not occur in humans (urinary bladder mesenchymal lesion and rat mononuclear cell leukemia). Moreover, for the most part, endocrine tissues, other than those related to estrogen, are not relevant to human cancer risk, such as the thyroid system, gastrointestinal tract, neuroendocrine tumors, and others. I have also listed tumors such as mouse lung (61), lymphoma (51, 52), and liver (60, 62), and rat pancreas (63–66) for which considerable evidence supports the conclusion that these are not relevant to human cancer risk. Although evaluations for the potential toxicity of these tissues are important, evaluation in rodents for the prediction of carcinogenesis in humans turns out not to be relevant.

**Table 1:** Rodent tumors not relevant to humans.

• Rodent organs without human counterpart
▪ Zymbal’s gland
▪ Harderian gland
▪ Forestomach
• Rodent tumors without human analog
▪ Rat splenic mononuclear cell leukemia
▪ Mouse submucosal mesenchymal lesion of bladder (seminal vesicles, uterus)
• Reproductive endocrine tumors
▪ Ovary—glanulosa cell
▪ Testes—Leydig cell (peritoneal mesothelioma)
▪ Endometrium
▪ Prostate
• Endocrine organs
▪ Thyroid
▪ Adrenal cortex
▪ Adrenal medulla
▪ Pituitary—anterior
▪ Pituitary—posterior
▪ Parathyroid
▪ GI neuroendocrine cells
▪ Pancreatic islets
• Questionable relevance
▪ Mouse lung
▪ Mouse liver
▪ Mouse lymphoma
▪ Rat pancreas

For *in vitro* assays, the major issue is what concentrations are to be evaluated, in addition to the issues of metabolic activation, what cells to be evaluated, and other issues described above. Essentially, all chemicals will be cytotoxic *in vitro* if a high-enough concentration is utilized. This is meaningless. Setting limits on concentrations to be used for *in vitro* assays as described above is essential.

Progress is being made in the development of *in vitro* assays for screening for carcinogens, and this can be utilized in conjunction with shorter-term animal bioassay (1–13 weeks). Combined with the suggested assays described above, this will provide an adequate screen for chemical carcinogenicity with respect to humans (1, 2, 4, 60). A major difficulty in validating new approach methods is as to what basis they are to be compared. Clearly evaluating assays for chemical carcinogenesis based on results in 2-year rodent bioassays is inappropriate since many of the results in the rodent are not relevant to humans. The focus has to be on screening for human carcinogenicity, not rodent carcinogenicity. In addition to the issues raised above with regard to the utilization of *in vitro* assays, there are other considerations. One of these is the reality that there are numerous cellular repair mechanisms available in tissues that protect us from the development of not only carcinogenicity but of toxicity. These have to be evaluated *in vitro* if these *in vitro* systems are to be used for screening for various toxicities, including carcinogenicity. Interactions of multiple cell types in a tissue are also critical and need to be evaluated. To some extent, these issues are being addressed utilizing 3D cultures, but these need considerable additional development before they can be utilized more broadly. They certainly can be used already to address specific biologic questions.

## Conclusions

Most scientists now agree that the long-term bioassay in rodents is no longer appropriate for screening for human carcinogenicity risk. Many agencies have already stopped performing such studies, such as the US National Toxicology Program. Various regulatory agencies are moving toward abandoning the requirement for a 2-year bioassay, but additional effort is necessary. Most importantly, changes in approach by regulatory agencies will require changes in guidelines, and even laws, for certain types of chemicals such as pharmaceuticals, agrochemicals, cosmetics, food ingredients, and other types of chemicals.

In this paper, I have presented a proposed approach for screening for chemical carcinogens based on mode of action, with a realization that only four basic modes of action are relevant to human carcinogens: DNA reactivity and mutagenesis, immunomodulation, increased estrogenic activity, and cytotoxicity with consequent regeneration. All but DNA reactivity are based on evaluations related to increased cell proliferation. Evaluation for these affects can be performed using alternative assays, including *in vitro*, *in silico*, and *in vivo*. Some basic questions regarding *in vitro* remain, such as metabolic activation, cell types to be evaluated, and concentrations to be utilized. Extrapolation to the *in vivo* situation is essential, with increasing progress being made on *in vitro* to *in vivo* extrapolation being accomplished by utilizing physiologically based pharmacokinetic (PPBK) models. The proposed approach for screening for carcinogenicity will be less costly, take less time, require fewer animals, and be more relevant to human risk than the use of the two-year bioassay in rodents. Of course, if an assay shows a positive signal, more extensive dose response analyses will be required to evaluate the actual risk at human exposures.

## Data availability statement

The original contributions presented in the study are included in the article/supplementary material. Further inquiries can be directed to the corresponding author.

## Ethics statement

The animal study was approved by Institutional Animal Care and Use Committee (IACUC). The study was conducted in accordance with the local legislation and institutional requirements.

## Author contributions

SC: Writing – review & editing, Writing – original draft.
